# The correlation between extracellular resistance by electrical biopsy and the ratio of optical low staining area in irradiated intestinal tissues of rats

**DOI:** 10.1186/1475-925X-12-23

**Published:** 2013-03-19

**Authors:** Yu-Jie Huang, Eng-Yen Huang, Kuo-Sheng Cheng

**Affiliations:** 1 Department of Biomedical Engineering, National Cheng Kung University, Tainan, Taiwan; 2 Department of Radiation Oncology, Kaohsiung Chang Gung Memorial Hospital and Chang Gung University College of Medicine, Kaohsiung, Taiwan; 3 Medical Device Innovation Center, National Cheng Kung University, Tainan, Taiwan

**Keywords:** Electrical impedance spectroscopy, Electrical biopsy, Radiation enteropathy, Hue-saturation-density (HSD) transformation, Ratio of low staining area, Extracellular resistance

## Abstract

**Background:**

Electrical biopsy illustrates a tissue’s electrical properties by electrical impedance spectroscopy. However, electrical biopsy parameters are different from conventional morphological-based examinations. The correlation between electrical biopsy and the morphological observation has not been checked. Considering the tissue responses to injury, extracellular resistance should be most sensitive with the accumulation of fluid in tissue, and it is expected to increase the ratio of optical low staining area on histological images. In this study, we calculated the ratio of optical low staining area of sampled histological images and compared with the results of electrical biopsy to verify the hypothesis of that the extracellular resistance of electrical biopsy most highly correlates with the ratio of optical low staining area on histological images.

**Methods:**

The irradiated intestinal tissues of rats after different latent period were used for study. The sampled tissues were measured by electrical impedance spectroscopy for electrical biopsy and the microscopic images were acquired. The sampled histological images were transformed into the Hue-Saturation-Density (HSD) colour model to decouple the stain density. The ratio of optical low staining area on histological images was computed to quantify the morphological changes. The results were related to the parameters from electrical biopsy according to three element circuit model by Spearman’s rank correlation test.

**Results:**

The ratio of optical low staining area varied as well as the tissue’s electrical parameters. The extracellular resistance (R_e_) and intracellular resistance (R_i_) by electrical biopsy tended to increase with the ratio of low staining area decreasing. The membrane capacitance (C_m_) by electrical biopsy tended to increase with the ratio of optical low staining area increasing. The extracellular resistance (R_e_) of electrical biopsy was the parameter most highly correlated with the ratio of optical low staining area with a correlation coefficient of −0.757 (p < 0.001).

**Conclusions:**

The results of this report confirm the hypothesis and support the idea that electrical biopsy results reflect the changes in tissues seen in conventional histological findings in a sense of conventional histological knowledge, and this approach may have a great potential for augmenting the pathological diagnosis of tissues.

## Background

Electrical impedance is one of the most often used parameters for characterizing material properties. The electrical impedance of a tissue is highly correlated with biological structure, including cell size, density, spacing, and the constituents of the extracellular and intracellular matrix [1]. Electrical impedance spectroscopy, revealing the variations in electrical impedance with changes infrequency, is good for obtaining both the resistive and capacitive characteristics of tissues. Schwan described the electrical properties of tissues and cell suspensions and concluded that electrical impedance analysis is a powerful research tool for biological applications [2]. Electrical impedance is a good marker for characterizing a tissue’s pathological changes, such as ischemia and neoplasms, both in human and animal subjects [3-12]. Electrical impedance spectroscopy of tissues has great potential for use in pathological analysis as an alternative or adjunct to the conventional morphological and histological examinations. This analysis is referred to as an “electrical biopsy”.

**Figure 1:**
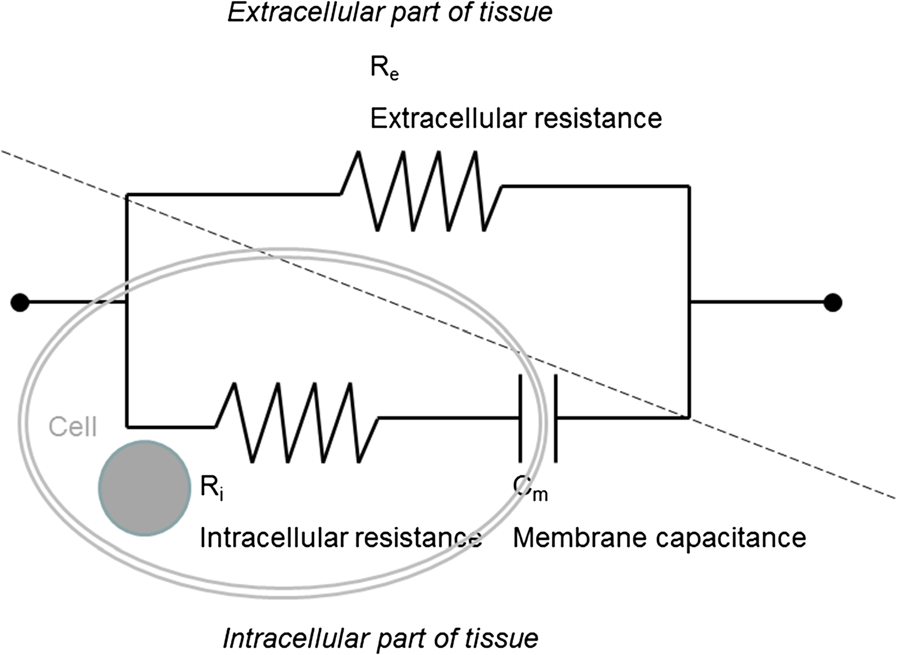
**The simplified equivalent three**-**element electrical circuit model for tissues.** The simplified equivalent three-element model to interpret the electrical properties of biological tissues, with R_e_ (extracellular resistance), R_i_ (intracellular resistance), and C_m_ (membrane capacitance).

The most well-known model for representing a tissue’s electrical characteristics is the three-element model depicted in Figure 1. It is a simple and easy model to understand from a biological aspect. This model was reported to be a good representation of the electrical properties of biological materials due to relaxation phenomena throughout the frequency range [2]. The simplified equivalent three-element model was applied for the interpretation of the electrical properties of biological tissues, with R_e_ (extracellular resistance), R_i_ (intracellular resistance), and C_m_ (membrane capacitance). R_e_ is contributed by the extracellular matrix (which is made up of mostly water), R_i_ by intracellular complex substances of the cytoplasm, and C_m_ by changes in cell membranes.

Considering the tissue’s responses to injury, edema is the most general histological reaction to injury. It is characterized fluid accumulation in tissues and presented as clearing and separation of the extracellular matrix [13]. It is supposed to be observed as increased areas of low staining and separation in the microscopic images of tissue. Based on this rationale, the ratio of optical low staining area of tissues on histological images could be used to quantify the tissue fluid status morphologically. Comparing the parameters of electrical biopsy to the ratio of optical low staining area in tissues, extracellular and intracellular resistance should be most sensitive with the accumulation of fluid in tissue, and the fluid in tissue is expected to increase the ratio of optical low staining area on histological images. However, intracellular cytoplasm is denser than extracellular space. The optical low staining area should mostly be contributed by extracellular space and its water content. Therefore, the R_e_ of electrical biopsy should most highly correlate with the ratio of optical low staining area on histological images by this rationale.

In this study, we established the algorithm to calculate the ratio of optical low staining area of sampled histological images from irradiated intestinal tissue in rats and compared with the results of electrical biopsy. The purpose is to verify the hypothesis of that the extracellular resistance of electrical biopsy most highly correlates with the ratio of optical low staining area on histological images.

## Methods

### Animal care and irradiated intestinal tissues

The radiation enteropathy of the rats was designed for this study. The specific pathogen-free Sprague–Dawley rats (males, weighing 300–350 g) were used in the experiments. There were four rats in each group. The control group was treated the same as the experimental group, except for the whole abdomen irradiation. An 18-Gy dose to the whole abdomen was applied, and the experimental groups were sacrificed at 3, 9, 14, 21, 28, 35, and 49 days after irradiation (annotated as D3, D9, D14, D21, D28, D35, and D49, respectively) for electrical biopsy and histological examinations of the intestinal tissues. All procedures and measurements were performed in strict accordance with protocols approved by the Chang Gung Memorial Hospital Animal Care and Use Committee.

### The electrical biopsy

**Figure 2:**
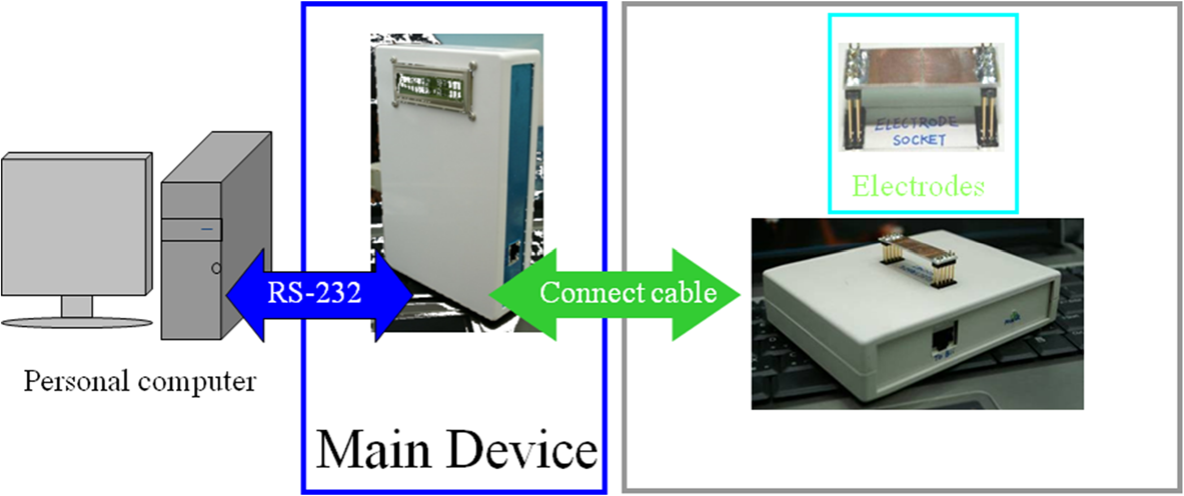
**The electrical impedance spectroscopy system for electrical biopsy.** The system is designed around an electrical impedance converter chip. The electrical impedance is measured with the tissues on electrodes. The data is transfer to personal computer to save and solve the electrical properties of tissues.

The electrical impedance spectroscopy system for electrical biopsy is designed around the electrical impedance converter chip (AD5933) acquired from Analog Devices (Norwood, MA, USA) and controlled by personal computer [14]. The system is showed on Figure 2. The system provided a frequency sweep mode for scanning electrical impedance values over a frequency band from 10 kHz to 100 kHz with 1 kHz steps. After the rat was sacrificed, a 5-cm length of distal jejunum, traced from the cecum was sampled. The tissue was dissected to expand a 5 × 5 mm sample. The specimen was placed on the electrodes with the mucosal surface down. The electrode were designed based on the two-electrode measurement method and were fabricated on a printed circuit board with two parallel plates of copper. The size of the electrode was the same as a glass slide for convenient tissue specimen study. The gap between the two electrode plates was 0.2 mm, with a saw line. The length of saw line is longer than a straight line. Therefore, the area of the saw line gap between electrodes is more effective for measurement. A cover glass was used to cover the specimen with a weight load of 20 g. The electrical impedance spectroscopy by the system was measured for electrical biopsy. The electrical parameters of R_e_, R_i_, and C_m_ were solved by ZSimpWin Version 3.1 (Princeton Applied Research, Oak Ridge, TN, USA) according to the impedance spectroscopy data. The impedance data from three-element RC model simulation with different scanning frequency range had been inputted to ZSimpWin to validate its ability and the adequacy of scanning frequency range. The results certify an excellent capability of ZSimpWin to solve the circuit model as Figure 1 with scanning frequency from 10 kHz to 100 kHz. The measured sample was then prepared for histological examination.

### Histological examination and image sampling

**Figure 3:**
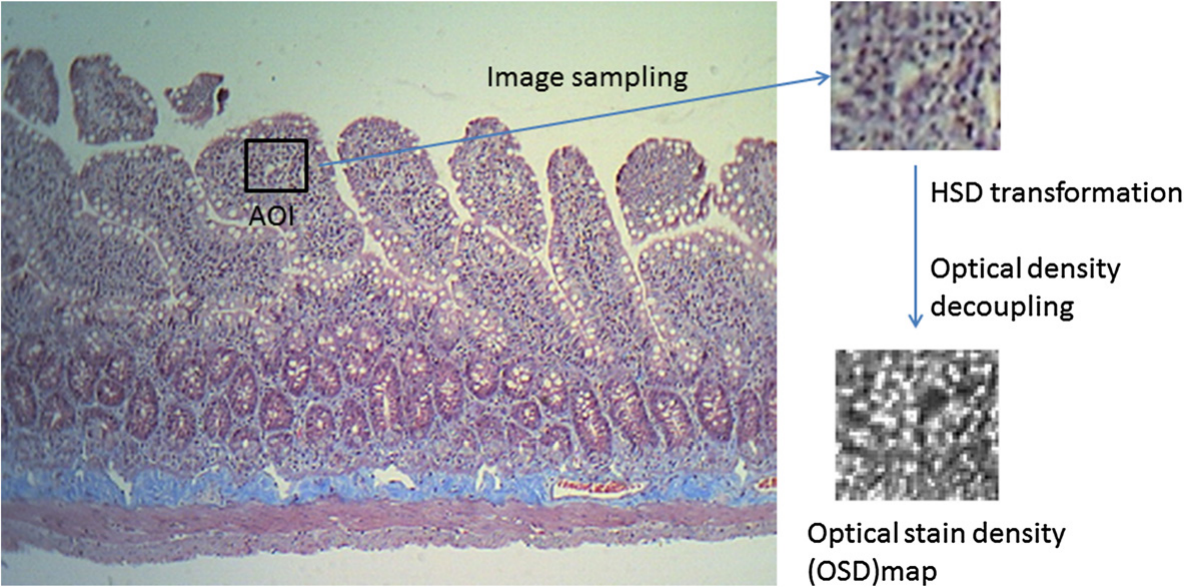
**The selected area of interest** (**AOI**) **and HSD transformation.** Diagram of the selected area of interest (AOI) and HSD transformation for computing the optical stain density (OSD).

After electrical biopsy, the specimens underwent histological examination using Masson’s trichrome stain. The histological images were acquired in digital form for later analysis using a transmitted light microscope at 100x magnification. For each specimen, four areas of interest (AOI) were selected for computing the optical low staining area. The AOI was defined as a maximum rectangle in mucosa and submucosa area, including lamina propria, and excluding air space of the villi within the histological images as illustrated in Figure 3.

### Computing the ratio of low staining area on histological images

The Hue-Saturation-Density (HSD) colour model was applied for staining area extraction of the images [15]. The monochromatic light travelling through an absorbing medium is in accordance with Lambert-Beers’ law of absorption [16]. Thus, the relationship between the intensity of monochromatic light transmitted through a specimen and the amount of stain present in the specimen may be represented as:

(1) $$I\left(\lambda \right)=I_{0}\left(\lambda \right)\;e^{-A\;c\left(\lambda \right)}$$

Where *I*_0_(λ) is the intensity of light of wavelength λ passing through the specimen, and *I*(λ) is the intensity of the light transmitted through the specimen. A is the quantity of stain per unit area of the specimen, and *c*(λ) is the function for the fraction of the incident light of wavelength λ transmitting through the stain.

The colour images acquired from the optical microscope are represented in pixels with colours in red, green, and blue (RGB). The sensitivity curve of the camera used for image acquisition is defined as the relationship between the incident light and the electric output signal. The output intensity of the camera for channel *ch* is represented by

(2) $$I_{\text{ch}}=\int_{0}^{\infty }S_{\text{ch}}\left(\lambda \right)\;I_{0}\left(\lambda \right)\;e^{-A\;c\left(\lambda \right)}\mathrm{d\lambda}$$

where *S*_*ch*_(λ) is the sensitivity of channel *ch* at wavelength λ. The filters used in the microscope camera were assumed to be narrow band-pass filters in which the sensitivity was restricted to the central frequency (=λ_*ch*_, where *ch* is R, G, or B); therefore, we have

(3) $$S_{\text{ch}}\left(\lambda \right)=\{\begin{array}{l} 1\;\text{if}\text{λ}=\lambda_{\text{ch}}\\ 0\;\text{if}\text{λ}\neq \lambda_{\text{ch}} \end{array}\;\}$$

Equation 2 can be simplified using equation 3 as

(4) $$I_{\text{ch}}=I_{0,\text{ch}}\;e^{-A\;c_{\text{ch}}}$$

where I_0,*ch*_ is the intensity of channel *ch* when no stain is present and *c*_*ch*_ is the absorption coefficient for λ = λ_*ch*_.

Therefore, the optical density (OD) of a channel *D*_*ch*_ depending linearly on the amount of stain could be defined as the absorption value of the staining at channel *ch*. It can be computed as

(5) $$D_{\text{ch}}=-\ln\left(\frac{I_{\text{ch}}}{I_{0,\text{ch}}}\right)=A\;c_{\text{ch}}$$

An overall measure for the OD can be further obtained from the following equation as

(6) $$D=\frac{D_{R}+D_{G}+D_{B}}{3}=\frac{A\;\left(c_{R}+c_{G}+c_{B}\right)}{3}$$

The overall OD is proportional to the staining per unit area of the specimen (*A*). Therefore, the optical stain density (OSD) of the sampled image (as shown in Figure 3) can be decoupled.

Then we define the low staining area is the area of lower 10% OSD on images. The ratio of low staining area is illustrated as

(7) $$\text{Ratio}\;\text{of}\;\text{optical}\;\text{low}\;\text{staining}\;\text{area}=\frac{\text{Area}\;\text{of}\;\text{lower}\;10\%\;\text{OSD}\;\text{on}\;\text{sampled}\;\text{image}}{\text{Total}\;\text{sampler}\;\text{image}\;\text{area}}$$

The value of the ratio of low staining area on sampled image for the tissue specimens could then be computed. The ratios of low staging area of four sampled AOI on each histological image were averaged for the result.

### Statistics

The Spearman’s rank correlation coefficient was applied to test the correlations between the results of electrical biopsy and the ratio of low staining area on sampled images. A *p*-value <0.05 was taken as statistically significant.

## Results

### The results of electrical biopsy

**Figure 4:**
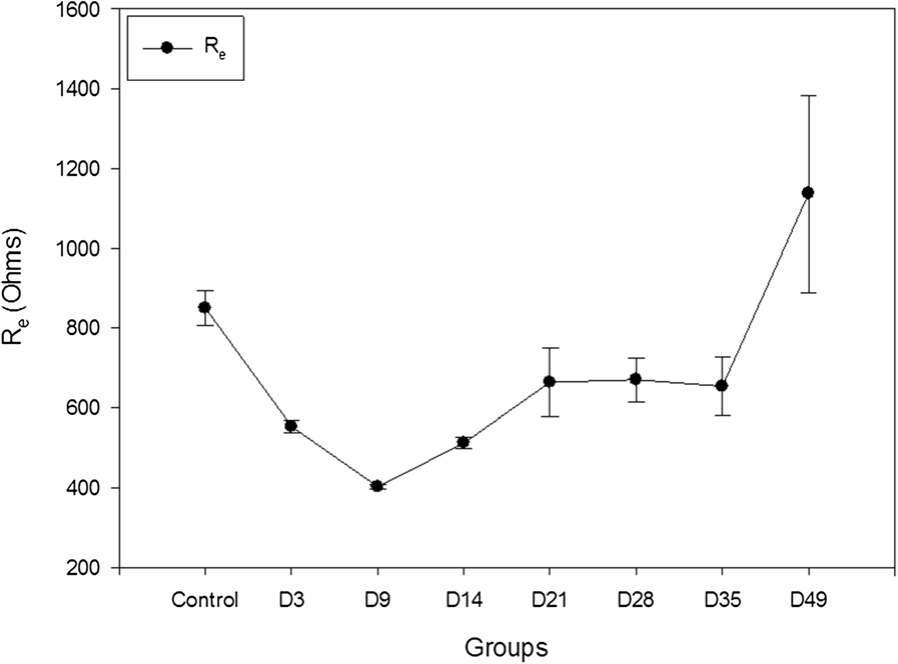
**The results of extracellular resistance in electrical biopsy according to experiment groups.** The mean value with bar of the standard deviation (SD) for extracellular resistance (R_e_) according to experiment groups.

**Figure 5:**
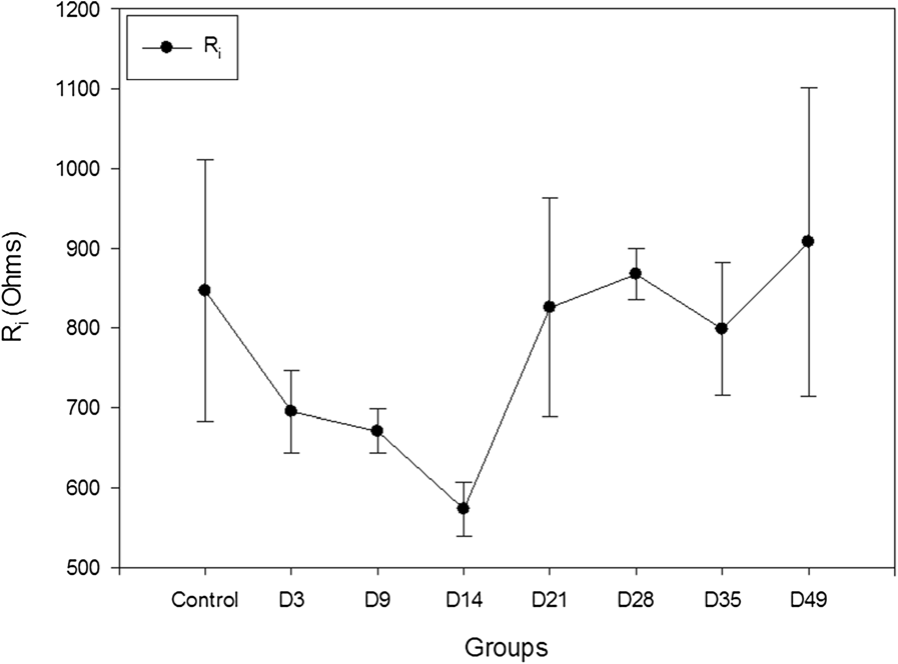
**The results of intracellular resistance in electrical biopsy according to experiment groups.** The mean value with bar of the SD for intracellular resistance (R_i_) according to experiment groups.

**Figure 6:**
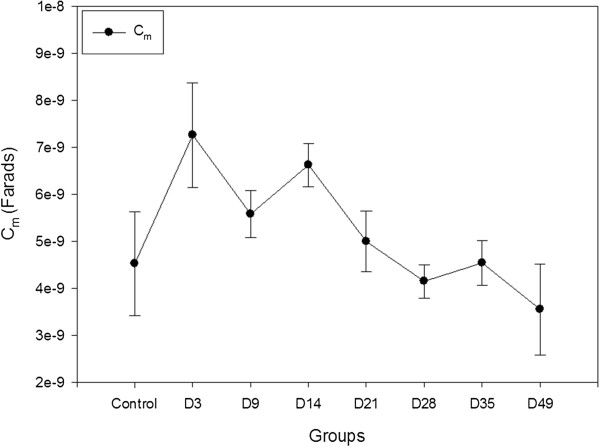
**The results of membrane capacitance in electrical biopsy according to experiment groups.** The mean value with bar of the SD for membrane capacitance (C_m_) according to experiment groups.

The results of electrical biopsy based on the parameters of the three-element RC electrical circuit model for each experimental group, as illustrated in Figures 4, 5, 6. The range of R_e_ was between 398 Ohms and 1375 Ohms with minimum in D9 group and maximum in D49 group. The range of R_i_ was between 529 Ohms and 1086 Ohms with minimum in D14 group and maximum in D49 group. The range of C_m_ was between 2.93*10^-9^ Farads and 7.26*10^-9^ Farads with minimum in D49 group and maximum in D14 group.

### The results for the ratio of low staining area

**Figure 7:**
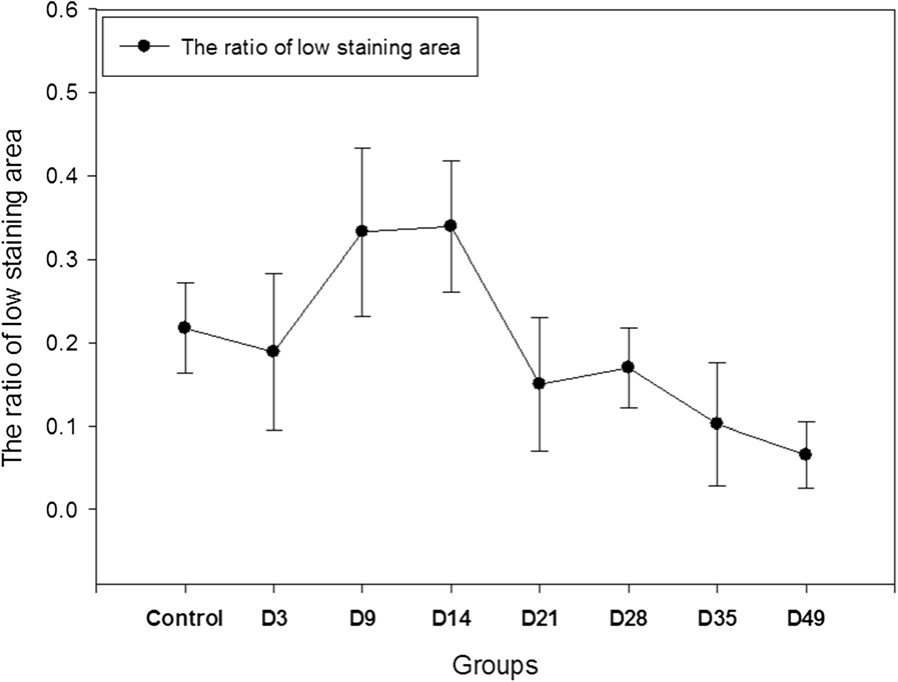
**The results for the ratio of optical low staining area according to experiment groups.** The mean value for the ratio of optical low staining area according to experimental groups, and the error bar indicating the standard deviation (SD).

The range was from 0.00264 to 0.49621 for the ratio of optical low staining area. The maximum was in D49 group and the minimum was in D9 group. Figure 7 shows the line plot with standard deviation bars for the experimental groups.

### The correlation between the ratio of optical low staining area and the parameters of electrical biopsy

**Figure 8:**
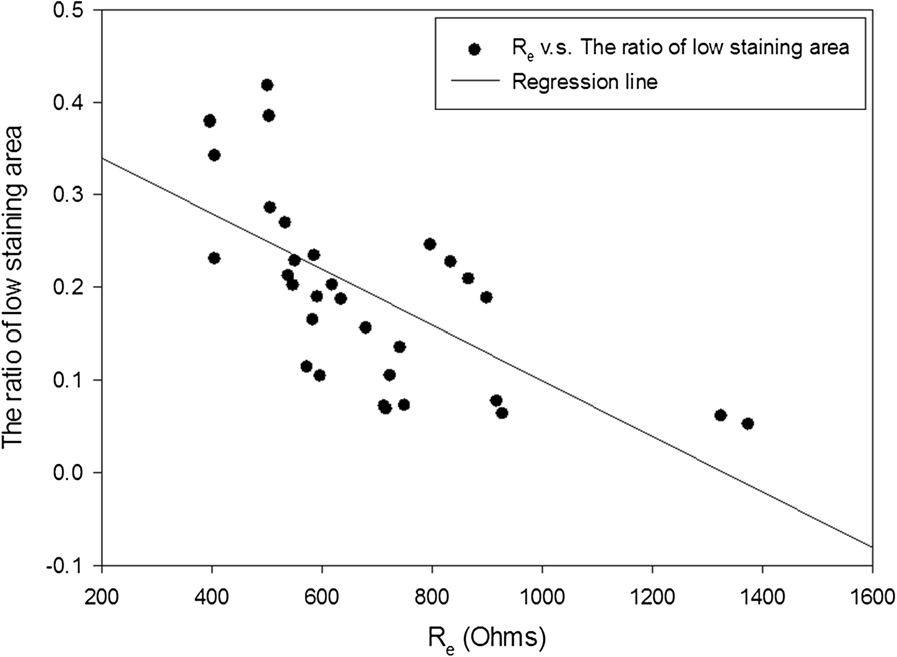
**The scatter plots for the extracellular resistance versus the ratio of low staining area.** The scatter and linear regression line plot for the value of extracellular resistance (R_e_) versus the ratio of optical low staining area.

**Figure 9:**
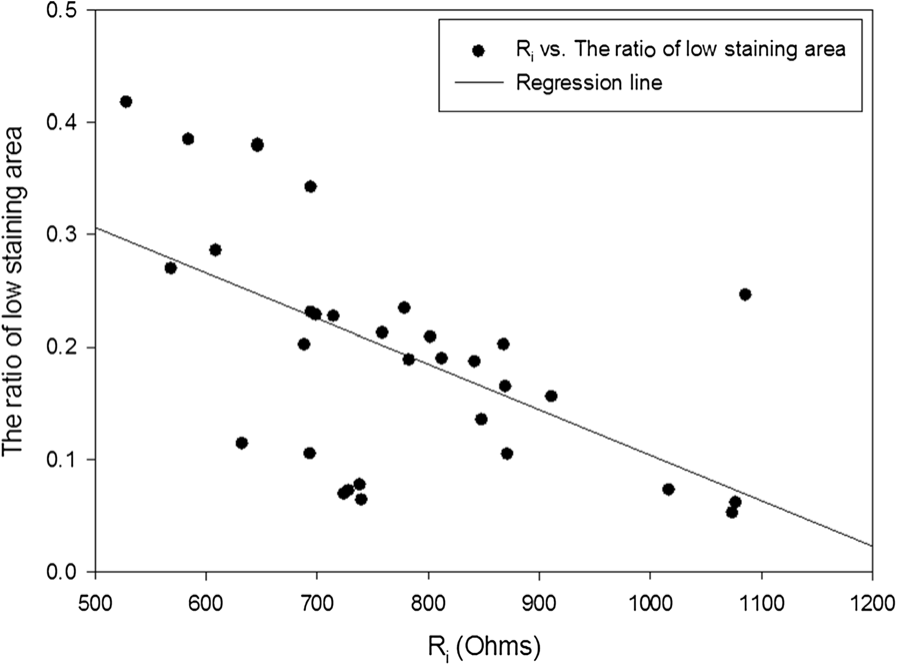
**The scatter plots for the intracellular resistance versus the ratio of low staining area.** The scatter and linear regression line plot for the value of intracellular resistance (R_i_) versus the ratio of optical low staining area.

**Figure 10:**
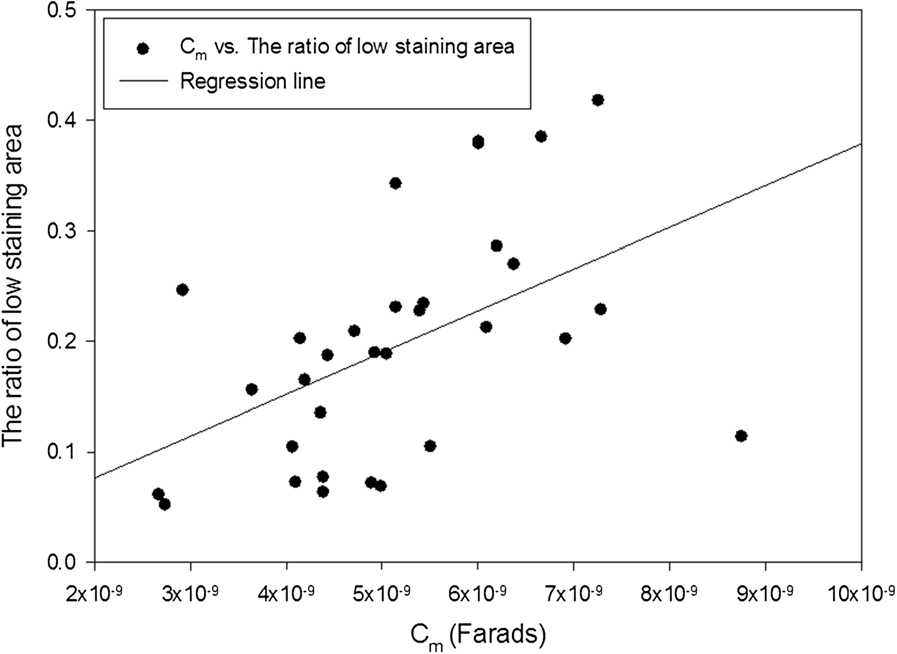
**The scatter plots for the membrane capacitance versus the ratio of low staining area.** The scatter and linear regression line plot for the value of membrane capacitance (C_m_) versus the ratio of optical low staining area.

Scatter plots with linear regression for the electrical parameter values of the electrical biopsy versus the ratio of optical low staining areas are illustrated in Figures 8, 9, 10. The extracellular resistance (R_e_) and intracellular resistance (R_i_) tended to increase with the ratio of low staining area decreasing. The membrane capacitance (C_m_) tended to increase with the ratio of optical low staining area increasing.

Table 1 shows the Spearman’s rank correlation coefficient between the electrical parameters obtained from the three-element model by electrical biopsy and the ratio of optical low staining area in the tissues. The most highly correlated parameter of electrical biopsy to the ratio of optical low staining area was extracellular resistance (R_e_), with a correlation coefficient of −0.757 (*p* < 0.001).

**Table 1:** The correlation between electrical parameters and the ratio of optical low staining area

**Correlations (95% Confidence Interval) *p-*** **value**	**The ratio of optical low staining area**	**R**_**e**_	**R**_**i**_	**C**_**m**_
**The ratio of optical low staining area**	**1**	**-**	**-**	**-**
R_e_	−0.757	1	-	-
	(−0.909 to −0.511)			
	*p* < 0.001^*^			
R_i_	−0.572	0.667	1	-
	(−0.825 to −0.218)	(0.407 to 0.815)		
	*p* < 0.001^*^	*p* < 0.001^*^		
C_m_	0.608	−0.680	−0.905	1
	(0.325 to 0.821)	(−0.799 to −0.494)	(−0.936 to −0.764)	
	*p* < 0.001^*^	*p* < 0.001^*^	*p* < 0.001^*^	

Spearman’s rank correlation coefficient between the electrical parameters from the three-element model by electrical biopsy and the ratio of optical low staining area in the tissues.

^*^p < 0.05.

## Discussion

The histological changes in the gastrointestinal epithelium after ionizing radiation are divided into two stages, an early stage and late stage [17]. Early radiation enteropathy is the damage of the intestinal mucosa and the first morphologic change after irradiation in this stage is serosa thickening. This is because of the extracellular fluid accumulation by tissue injury. Subsequently, late radiation enteropathy occurs after a variable latency. The manifestations of late radiation enteropathy are considered to be vascular and connective tissue damage. These phenomena in late phase frequently occur in the intestinal wall, instead of the mucosa, with vascular sclerosis and intestinal wall fibrosis [18]. The occurrence of vascular sclerosis and intestinal fibrosis is likely to decrease extracellular fluid.

According to the tissue’s electrical parameters with the three-element RC equivalent electrical model in Figure 1, R_e_ is extracellular resistance that should imply the status of the extracellular fluid. For the fluid in biological tissue is conductive, the R_e_ should decrease with the extracellular fluid increasing. As our results, R_e_ was decreased after irradiation up to day 9 in early phase for tissue fluid accumulation, and increased in day 49 for fibrosis in late phase rationally (Figure 4). The other two electrical parameters of electrical biopsy, R_i_ and C_m_, demonstrated the similar response to tissue injury for their representation of changes in tissues (Figures 5 and 6). Therefore, electrical biopsy may be a quantified method to evaluate the tissue’s status.

Conventional histological examinations involve morphological examination under a light microscope. These rely on the human eye and depend on the experience of the observer. Although some criteria or descriptions are used to evaluate the histological morphology, they are subjective and not quantitative. The judgment of the tissue status by morphological examination varies individually. It is the same in evaluation of the fluid status in tissues. Edema denotes an excess of fluid in the interstitial or serous cavities and is characterized as clearing and separation of the extracellular matrix in textbooks [13]. The assessment of the fluid status of tissue, i.e. edema, is descriptive in conventional way.

For quantifying the fluid status of tissue on histology images, the descriptions for evaluate the tissue’s fluid status should be defined quantitatively. This clearing of extracellular matrix should be observed as low staining, and the separation should be defined as increasing areas on histological images. For calculating the amount of stain on the histological image, the chromatic information should be decoupled. RGB intensities are the most often used colour space that is closest to the way of human eye. However, the colour of the stain is variable, and algorithms are needed to perform classification in the RGB space for each channel. To avoid the use of complicated algorithms, the RGB model can be processed via monochromatic threshold in separate channels or by projection onto a plane or a line [19-24]. For evaluating the stained histological images, projection the colour onto a line or plane for analysis could be a good solution.

The most popular method used to extract the chromatic information from the RGB images is the hue-saturation-intensity (HSI) model [25]. The HSI transformation from the RGB model decouples the intensity information from the colour information. The intensities are independent with a linear relation to channel intensity. However, according to equation 1, the optical intensities show a logarithmic relationship (as opposed to linear) with the amount of staining. Therefore, the HSD model is adapted from the HSI model with optical densities converted from intensities. The optical densities are linear relative to the amount of staining (as in equation 5), which is linear to the amount of stain. The optical density of the HSD model is independent of the chromatic component and, therefore, offers a good representation of the amount of stain.

Following the accumulation of fluid in tissues, the substances that can be stained are separated and diluted. Thus, the low staining area in the histological images increases. We supposed that the low staining is the optical density less than 10%. The proportion of optical low staining area in the sampled histological image should be a good index for the fluid status of tissue. The ratio of optical low staining area is hence defined as in equation 7. In this study, the ratio of optical low staining area was elevated after day 3 and decreased relative to the control after day 21 (Figure 7). This was in accordance with the morphological description of radiation enteropathy [17].

Comparing the values of the electrical parameters of electrical biopsy and the ratio of optical low staining area on histological images, both extracellular and intracellular electrical resistance of the tissues tended to decrease with the ratio of optical low staining area increasing (Figures 8 and 9). This is reasonable for the increased conductivity by the fluid accumulation both extracellularly and intracelluarly. The membrane capacitance (C_m_) tended to increase with increasing ratio of optical low staining area (Figure 10). This may be due to the decrease in membrane permeability (resulting in increasing capacitance) that occurs to prevent cell swelling following extracellular fluid accumulation.

Most stained substances are located in the cytoplasm; therefore, the ratio of optical low staining area is primarily a reflection of extracellular status. According to this rationale for tissue fluid status evaluation in electrical biopsy and optical low staining area rating, the R_e_ and ratio of optical low staining area should be the most highly correlating parameters for extracellular fluid status. This hypothesis is tested on Table 1 with a confirmed result. Because the electrical parameters of the tissue are not only impacted by water content but also by any other tissue characteristics, the correlations were not particularly high. However, R_e_ was indeed the most correlative parameter relative to the ratio of optical low staining area based on Spearman’s rank test with a correlation coefficient of −0.757, and a *p*-value <0.001.

Electrical biopsy is a novel method for augmenting pathological diagnosis and screening of tissue status. However, the results of the electrical biopsy provide electrical parameters that are different from those conventional morphological-based histological examinations. Most of the identification of the histological change is under microscopy by morphology due to this is the most conventional and experienced way for tissue examination. The electrical biopsy may be applied to extend the histological examination for quantifying the tissue’s characteristics in addition to the observed morphological variations. In this study, we evaluated the ratio of optical low staining area on histological images to correlate the parameters of the electrical biopsy. It was shown that the extracellular resistance was the most highly correlated parameter. This implied that the electrical biopsy indeed reflected changes of the tissue corresponding to conventional morphological findings in a sense of conventional histological knowledge. Therefore, the electrical biopsy may have a great potential for augmenting the pathological diagnosis of tissues. However, these results is still primitively, further experiments should be designed to check the correlation between the electrical parameters and other morphological changes observed via conventional histological examination, in order to facilitate the application of the electrical biopsy.

## Conclusions

The extracellular resistance (R_e_) estimated using the concept of electrical biopsy was shown to be most highly correlated the ratio of low staining area on the sampled histological images because of extracellular fluid accumulation in tissue injury response. These results illustrate that electrical biopsy corresponds to the morphological changes in a sense of conventional histological knowledge. Therefore, The electrical biopsy reflects the histological changes and has demonstrated great potential for augmenting the pathological diagnosis of tissues.

## Competing interests

There is no competing interest associated with this study.

## Authors’ contributions

The contributions of the authors are as follows: YJH have made substantial contributions to conception and design, carried out the electrical biopsy, image analysis and interpretation of data, and drafting the manuscript. EYH participated in animal experiment and histological image acquisition. KSC was the advisor and coordinator of this study. He also helped to draft and revise the manuscript. All authors read and approved the final manuscript.
